# Mismatch repair gene mutations lead to lynch syndrome colorectal cancer in rhesus macaques

**DOI:** 10.18632/genesandcancer.170

**Published:** 2018-03

**Authors:** Beth K. Dray, Muthuswamy Raveendran, R. Alan Harris, Fernando Benavides, Stanton B. Gray, Carlos J. Perez, Mark J. McArthur, Lawrence E. Williams, Wallace B. Baze, Harsha Doddapaneni, Donna M. Muzny, Christian R. Abee, Jeffrey Rogers

**Affiliations:** ^1^ Michale E. Keeling Center for Comparative Medicine and Research, Department of Veterinary Sciences, University of Texas MD Anderson Cancer Center, Bastrop, Texas, USA; ^2^ Human Genome Sequencing Center and Dept. of Molecular and Human Genetics, Baylor College of Medicine, Houston, Texas, USA; ^3^ The Virginia Harris Cockrell Cancer Research Center, Department of Epigenetics and Molecular Carcinogenesis, University of Texas MD Anderson Cancer Center, Smithville, Texas, USA; ^4^ Department of Veterinary Medicine and Surgery, University of Texas MD Anderson Cancer Center, Houston, Texas, USA

**Keywords:** colorectal cancer, Lynch syndrome, rhesus macaque, MLH1, MSH6

## Abstract

Colorectal cancer accounts for a substantial number of deaths each year worldwide. Lynch Syndrome is a genetic form of colorectal cancer (CRC) caused by inherited mutations in DNA mismatch repair (MMR) genes. Although researchers have developed mouse models of Lynch Syndrome through targeted mutagenesis of MMR genes, the tumors that result differ in important ways from those in Lynch Syndrome patients. We identified 60 cases of CRC in rhesus macaques (*Macaca mulatta*) at our facility since 2001. The tumors occur at the ileocecal junction, cecum and proximal colon and display clinicopathologic features similar to human Lynch Syndrome. We conducted immunohistochemical analysis of CRC tumors from several rhesus macaques, finding they frequently lack expression of MLH1 and PMS2 proteins, both critical MMR proteins involved in Lynch Syndrome. We also found that most macaque cases we tested exhibit microsatellite instability, a defining feature of Lynch Syndrome. Whole genome sequencing of rhesus macaque CRC cases identified mutations in *MLH1* and/or *MSH6* that are predicted to disrupt protein function. We conclude that this population of rhesus macaques constitutes a spontaneous model of Lynch Syndrome, matching the human disease in several significant characteristics, including genetic risk factors that parallel human Lynch Syndrome.

## INTRODUCTION

Colorectal cancer (CRC) is a common, often fatal disease that ranks third in the United States and fourth in the world among all forms of cancer as a cause of death [1, 2]. The proportion of CRC cases attributable to genetic factors is estimated to be up to 30%, with approximately 5% caused by inherited mutations that significantly increase lifetime risk. Lynch syndrome is the most common heritable form of CRC and is transmitted as an autosomal dominant trait. This disease is characterized by an earlier age of onset, a more proximal location than most other forms of CRC, and has a prevalence of approximately one in 440 [3]. Histologically, Lynch syndrome CRCs are often mucinous, poorly differentiated, and have large numbers of tumor-infiltrating lymphocytes (TILs) as well as prominent peri-tumoral lymphoid aggregates, known as a Crohn's-like reaction [4]. Lynch syndrome is caused by damaging mutations in DNA mismatch repair genes that play important roles in the detection and repair of DNA mutations [5]. It is this predisposition to suffer the loss of critical DNA repair mechanisms that significantly increases the likelihood of cancer in Lynch syndrome patients.

In the past, the term Lynch syndrome has been used interchangeably with hereditary non-polyposis colorectal cancer (HNPCC); however, current practice favors the use of HNPCC to refer to a clinical diagnosis based on familial clustering and age of disease onset as defined by the Amsterdam or Bethesda criteria, while Lynch syndrome refers only to cases that fulfill those criteria and have identified mutations in DNA mismatch repair (MMR) genes [6, 7]. Specifically, Lynch syndrome is distinguished from other forms of HNPCC through the positive identification of mutations in one or more of four MMR genes: *MLH1, MSH2, MSH6* and *PMS2* [6]. The loss of function of one or more MMR genes is associated with both increased risk of cancer and elevated rates of mutation in other regions of the genome, not necessarily related to disease. For those diagnosed with mutations in MMR genes, the risk of developing colorectal cancer by age 70 is 35%, while the risk of endometrial cancer by age 70 is 34% and the ovarian cancer risk is 8% [5]. However, the likelihoods of colorectal and endometrial cancer differ depending on which MMR gene is mutated, so the risk for any individual patient may be higher than these figures. The risk for stomach or small bowel cancer is also elevated among MMR mutation carriers [5]. In addition, individuals with MMR mutations show increased rates of DNA replication errors in the mono-, di- or trinucleotide repeats that occur at thousands of locations across the human genome, so-called microsatellite loci [8]. Consequently, one hallmark of Lynch syndrome is microsatellite instability (MSI), a phenomenon in which somatic mutations increase or decrease the numbers of tandem repeat units present within individual microsatellite loci. These somatic mutations are easily recognized as variances in repeat unit number for any given microsatellite locus, detectable with routine molecular genetic testing [8, 9]. Furthermore, mutations that affect mononucleotide runs (MNR) or other short repeats present in the coding sequences of genes can produce frameshift mutations that alter protein sequence and may directly influence onset or progression of CRC [10]. Thus, MSI is one characteristic feature of Lynch syndrome and is widely used in diagnosis.

The recommended clinical management of patients with Lynch syndrome includes regular colonoscopy for early detection [3], and there is evidence that daily aspirin can reduce the risk of CRC in these patients [11]. However, there are many unresolved questions concerning Lynch syndrome, its causes, and the optimal clinical management. For example, the influence of other modifying genes (beyond the four primary MMR loci) on disease risk, age of onset or disease progression (e.g. metastases) remains a topic of active research. The mechanism underlying the therapeutic effect of aspirin has not been established [12], and the optimal dose remains unclear [3]. Importantly, recent advances suggest that immunotherapy may provide significant improvements in treatment options for Lynch Syndrome cancers, but these strategies are not yet routinely available to patients, and require additional development [10, 13].

Attempts to develop mouse models of Lynch syndrome have been partially successful, but suffer from important weaknesses. Unlike humans, mice that are heterozygous for loss-of-function mutations in *Mlh1*, *Msh2* or *Msh6* do not exhibit elevated risk for colorectal cancer [14]. Mice that are homozygous for such mutations do develop cancer, but the nature of the tumor burden and the specific distribution of tissues affected are different from human Lynch syndrome [12]. Thus, a well-validated and predictive animal model of Lynch syndrome is currently lacking. Most importantly, an appropriate animal model would provide new opportunities for controlled experimental comparison of alternative preventative or therapeutic regimes.

Aged rhesus macaques (*Macaca mulatta*) have a relatively high incidence of spontaneously occurring colorectal cancer [15-17]. The clinicopathologic features of CRC in rhesus monkeys resemble those described in patients with Lynch syndrome, but little is known about the cause of macaque cancers. CRC has been routinely diagnosed in aged rhesus macaques at the MD Anderson Cancer Center Keeling Center for Comparative Medicine and Research (KCCMR) over the past 17 years (60 cases between 2001-2017). Recent detailed analyses have shown that the clinical and pathological features of macaque CRC are often very similar to those of Lynch syndrome in humans. DNA sequence analysis of affected macaques revealed that many carry heterozygous germline mutations in *MLH1* and/or *MSH6*. Investigations of the identified pathology, levels of MMR protein expression and evidence for MSI demonstrate that these macaques more closely recapitulate human Lynch syndrome than do any existing animal models. Given their close phylogenetic position as Old World monkeys, rhesus macaques are genetically more similar to humans than are other widely used animal models [18] and thus provide translational models that share with humans more genetic, physiologic and pathophysiologic similarities than other laboratory species [19, 20]. We anticipate that this spontaneous primate model will prove valuable for novel experiments intended to address some of the unanswered questions regarding pathogenesis and management of colorectal and other cancers in human carriers of MMR mutations.

## RESULTS

### Clinicopathologic findings

CRC in the KCCMR rhesus macaques most commonly presents as chronic weight loss, sometimes associated with diarrhea. In the current cohort of 20 CRC animals, 15 had weight loss of greater than 10% over the preceding year and 10 had diarrhea on presentation (Table 1). The average age at diagnosis in this cohort was 18 years, which matches the overall mean age at diagnosis for all cases over the past 17 years, an age that is equivalent to the mid-50's in humans. Additionally, rhesus macaques with CRC are often anemic and hypoproteinemic. Within the current cohort, 12 out of 20 were diagnosed with anemia (defined as a hematocrit less than 35) and 17 were hypoproteinemic (total protein less than 6.5 g/dL). All 20 animals were diagnosed as hypoalbuminemic based on an albumin of less than 4.0 g/dL. In the 12 animals that were tested for occult blood in the feces, 8 were positive. Although fecal occult blood can be a good indicator of CRC in humans, rhesus macaques can often be positive with various infectious diarrheas, which are quite common in this species. Therefore the utility of fecal occult blood analysis is limited in the diagnosis of CRC in rhesus monkeys. On physical examination, a palpable abdominal mass was detected in 17 of the affected animals. Differential diagnoses sometimes included endometriosis or a gastrointestinal foreign body, although CRC was usually the top differential given by the clinical veterinarian.

**Table 1:** Signalment and clinical parameters in rhesus macaques with CRC

Animal ID	Sex	Age (years)	Percent weight loss	Diarrhea on presentation	Hct	Total protein (g/dL)	Albumin (g/dL)	Fecal Occult Blood	Mass palpated
J073	F	20	11	N	33.3	6.7	3.7	N	Y
H285	F	9	26.6	Y	34.4	5.3	2	Y	N
L893	F	22	27.7	N	32	5.7	2.2	ND	Y
J031	F	21	21.3	N	29.1	5.9	2.9	Y	Y
J536	M	17	16.9	N	28.6	5.1	3.2	Y	Y
J071	F	22	24.7	Y	31	5	2.2	Y	Y
J809	F	14	19.1	N	41	5.5	3.1	ND	Y
J469	F	18	32.5	N	30	5.9	2.8	N	Y
J340	M	21	4.1	Y	44.2	6.3	3.5	N	N
J735	F	16	8.9	N	33.8	5.1	2.5	Y	Y
J435	F	19	12.9	Y	36.5	5.3	2.5	N	N
J731	F	17	15.7	N	36.1	4.7	2	Y	Y
J851	F	17	18.5	Y	35.7	5.3	2.2	Y	Y
J453	F	21	9.5	N	20	6	2.6	ND	Y
J727	F	18	19.9	Y	43	6.6	2.6	ND	Y
J391	F	22	18.7	Y	34.8	5.3	2.2	Y	Y
J725	F	19	9.2	Y	26.1	3.8	2.3	ND	Y
5-101	F	11	19.6	Y	26.2	5.8	2.9	ND	Y
J343	F	24	4.1	N	45.5	6.7	3.1	ND	Y
J737	F	20	17.6	Y	42.1	5.2	2.8	ND	Y

N = no, Y = yes, ND = not done

**Figure 1:**
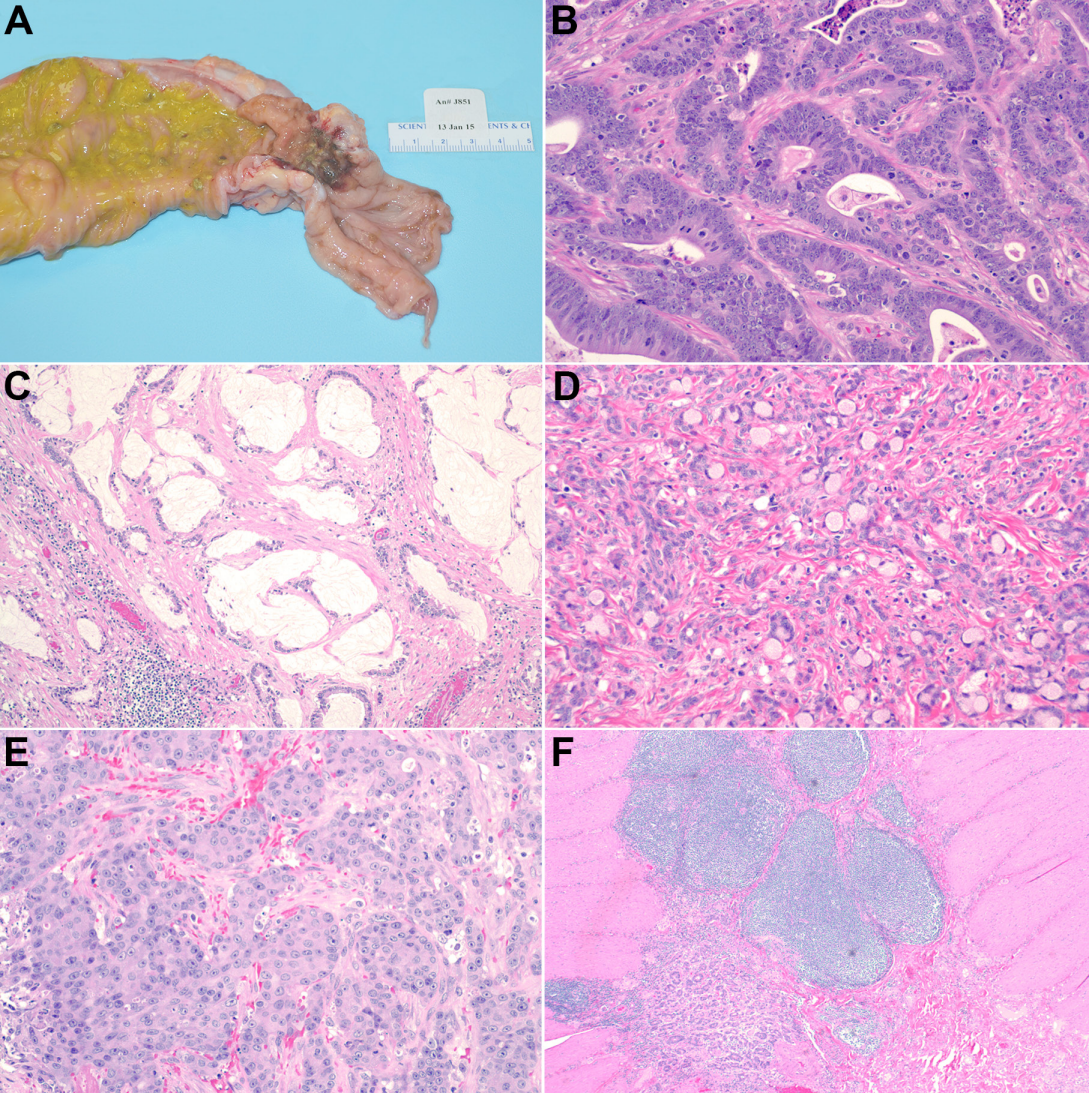
Gross and Histopathologic Features **A.** Gross appearance of tumor in animal J851 showing strictured, ulcerated mass in the proximal colon. **B.** Invasive, atypical glandular structures in J725, H&E, 100X. **C.** Mucinous differentiation in J727, H&E, 200X. **D.** Signet ring morphology in J851, H&E, 200X. **E.** Medullary carcinoma in J343, H&E, 200X. **F.** peri-tumoral lymphoid aggregates in J391, H&E, 40X.

At necropsy, all animals were found to have tumors at the ileocecal junction, cecum, or proximal colon (Table 2). In 15 of the 20 affected animals, there was a single tumor; however, in 5 animals there were 2 or more distinct tumors, all of which occurred between the ileocecal junction and the mid-colon. The tumors often had a strictured “napkin-ring” appearance grossly, with narrowing of the intestinal lumen and mucosal ulceration (Figure 1a). Microscopically the tumors were considered moderately to poorly differentiated and demonstrated invasion of the colonic wall by atypical glandular structures. Some tumors had mucinous differentiation with abundant extracellular accumulation, (Figure 1b) and/or signet ring morphology with intracellular mucin displacing the nucleus laterally (Figure 1c). Only 1 tumor demonstrated areas consistent with a medullary carcinoma (Figure 1d). The degree of invasion varied from those limited to the submucosa, to those that had penetrated the wall and spread to adjacent viscera. A characteristic desmoplastic response was seen in all tumors. So-called “dirty necrosis” could be observed in most tumors, but was generally not a conspicuous feature. TILs and a prominent peri-tumoral lymphocytic infiltrate, often forming nodular aggregates, was seen in all tumors (Figure 1e). Lymphatic, vascular, and perineural invasion was rare, but was seen in a limited number of cases (Table 2). Metastasis was documented in 6 cases; in 5 of those it was limited to the nearby mesenteric lymph nodes (Figure 1f), but in 1 case metastatic cells were seen microscopically on the surface of the diaphragm. Common co-morbidities included amyloidosis and osteoarthritis, likely attributable to the chronic inflammation seen in the tumors and the animal's advanced age, respectively.

**Table 2:** Pathologic features in rhesus macaque CRC

ID	Tumor location	Invasion	Lymphatic/vascular invasion	Peri-neural invasion	Metastasis	Mucinous	Signet ring	Medullary carcinoma	Peri-tumoral lymphocyte nodules
			+/−	+/−	(−) No (+) Local LN (++) Distant	−/+/++	−/+/++	+/−	−/+/++
J073	pc	T3	−	−	++	+	−	−	+
H285	3 : IC; cecum; pc	T3	+	+	+	−	−	−	++
L893	IC	T3	−	−	−	−	−	−	++
J031	IC	T4 (adjacent LN)	−	−	−	−	−	−	++
J536	4 : IC;cecum;2 pc	T2	−	+	−	−	−	−	++
J071	pc	T3	−	−	+	++	+	−	++
J809	IC	T4 (adjacent duodenum)	+	−	−	++	+	−	+
J469	IC	T3	−	−	−	−	−	−	++
J340	2 : pc; mid colon	T3	−	−	−	+	+	−	+
J735	IC	T3	−	−	−	+	−	−	+
J435	2 : IC and pc	T3	−	−	−	+	+	−	++
J731	pc	T2	−	−	−	−	−	−	++
J851	pc	T3	−	−	+	−	−	+	+
J453	IC	T3	−	−	+	−	−	−	+
J727	pc	T3	−	−	−	+	++	−	+
J391	pc	T3	−	−	−	−	−	−	++
J725	IC	T3	−	−	−	++	+	−	+
5-101	2 : IC and pc	T3	−	−	+	−	−	−	+
J343	IC	T2	−	−	−	−	−	−	++
J737	pc	T3	−	+	−	+	−	−	+

IC = ileocecal junction; pc = proximal colon; T1 = submucosa invasion, T2 = muscularis invasion, T3 = transmural invasion, T4 = adjacent invasion

### Immunohistochemical analyses of protein expression

**Figure 2:**
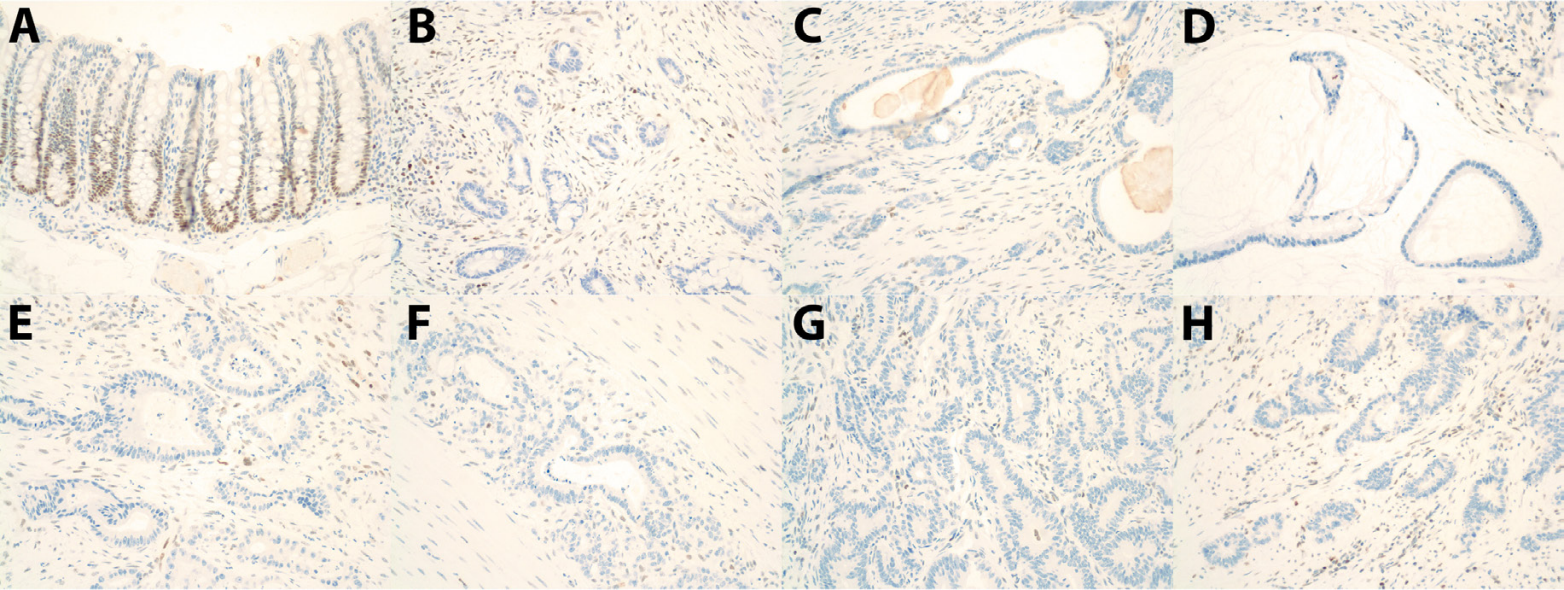
MLH1 Immunohistochemistry Results **A.** Normal colon from an unaffected rhesus macaque showing positive nuclear staining in the colonic epithelium. **B.-H.** Loss of staining in colorectal tumors from animals: J073, L893, J071, J469, J735, J731, and J391.

Immunohistochemical analysis of the 4 mismatch repair proteins implicated in Lynch syndrome (MLH1, PMS2, MSH6, and MSH2) revealed loss of MLH1 and PMS2 staining in the tumors from 17 of the 20 animals from this cohort (Figure 2). Tumor tissue from one animal retained strong staining for MLH1 and PMS2. The IHC in the 2 remaining animals was deemed equivocal due to the absence of staining in the internal positive control (lymphocytes and normal mucosal epithelium). MSH6 and MSH2 staining was retained in tumors from all animals except the 2 in which staining was equivocal.

### Microsatellite instability

We examined MSI in tumoral DNA versus normal DNA from 9 of the rhesus macaques diagnosed with CRC, all drawn from the primary cohort of 20 cases. These analyses used genotypes for twelve microsatellite loci (9 rhesus dinucleotide repeats and 3 human mononucleotide repeats). We observed that the DNA from 6 of these 9 tumors showed consistent microsatellite instability compared with DNA obtained from normal tissue from the same animal (Table 3). Among the six, three tumors showed low instability and three displayed high instability.

**Table 3:** Microsatellite analysis

Markers	L893	J031	J809	J340	J735	J435	J731	J851	J727
BAT24	MSS	MSS	MSS	MSS	MSS	MSS	MSS	MSS	MSS
BAT25	MSS	MSS	MSS	**MSI**	MSS	MSS	MSS	MSS	MSS
NR21	MSS	MSS	MSS	MSS	MSS	MSS	MSS	**MSI**	MSS
MML3S7	MSS	MSS	**MSI**	**MSI**	MSS	MSS	MSS	**MSI**	MSS
MML3S9	**MSI**	MSS	**MSI**	**MSI**	MSS	MSS	MSS	**MSI**	**MSI**
MML4S1	MSS	**MSI**	**MSI**	MSS	MSS	MSS	MSS	MSS	MSS
MML11S1	MSS	MSS	MSS	MSS	MSS	MSS	MSS	MSS	MSS
MML14S6	MSS	MSS	MSS	MSS	MSS	MSS	MSS	MSS	MSS
MML16S3	MSS	MSS	MSS	MSS	MSS	MSS	MSS	MSS	MSS
MML17S1	**MSI**	MSS	**MSI**	MSS	MSS	MSS	MSS	**MSI**	MSS
MML17S2	MSS	MSS	**MSI**	MSS	MSS	MSS	MSS	**MSI**	MSS
MML18S3	MSS	MSS	MSS	**MSI**	MSS	MSS	MSS	MSS	MSS
	**MSI-L**	**MSI-L**	**MSI-H**	**MSI-H**	**MSI-S**	**MSI-S**	**MSI-S**	**MSI-H**	**MSI-L**

MSI-stable (MSI-S), no markers showing instability, MSI-low (MSI-L), 1-2 markers showing instability, and MSI-high (MSI-H), 3 or more markers showing instability.

### Whole genome sequencing

To extend our analyses of the rhesus macaque CRC model, we performed whole genome sequencing on 20 CRC cases and 7 controls (confirmed no CRC by necropsy) from KCCMR. Across those 20 affected individuals, 8 were heterozygous and one homozygous for a 2-bp deletion in the promoter region of *MLH1* (Table 4). Four of those 9 individuals also had a *de novo* stop codon in the coding region of *MLH1*. This *de novo* stop codon occurs in exon 11 out of 19 in the *MLH1* gene, and thus would produce a dramatically truncated and possibly non-functional protein. Six of the 20 individuals we sequenced carried a heterozygous missense mutation in *MSH6* that has a CADD score of 22.8, indicating that it would be among the <1% most deleterious mutations possible in the human genome (if that variant occurred in the human *MSH6* gene). Furthermore, two of those six animals carrying the damaging missense mutation in *MSH6* also carried the 2-bp deletion in the *MLH1* promoter. Among the 7 controls from the KCCMR colony, we found no individuals with predicted damaging mutations in *MSH2, MSH6* or *PMS2*. We did find one of the controls was heterozygous for both the stop codon gained and the 2-bp deletion in the promotor of *MLH1*. This animal was found to be free of CRC at necropsy but was diagnosed with ocular carcinosarcoma. We also searched our database of whole genome sequences from 510 rhesus macaques selected randomly from the NIH National Primate Research Center colony populations, in order to assess the background frequency of these mutations in the general population of rhesus macaques in US research centers. We found no other individuals that were either heterozygous or homozygous for the *MLH1* de novo stop codon or the *MSH6* damaging missense mutation. Fast-LMM analyses of association between these two variants and the CRC/Lynch syndrome phenotype, using both the 510 undiagnosed rhesus macaques and the 7 confirmed unaffected as controls, show this is a very strong association (*p* < 3.4 × 10^−16^ and *p* < 1.9 × 10^−25^, respectively). The 2-bp deletion does occur in other research colonies. We found that the allele frequency of the 2-bp deletion is 0.44 across that set of 517 undiagnosed or unaffected rhesus macaques, making this a common polymorphism in this species. However, we unfortunately have no information regarding presence of CRC among the carriers of that deletion in those other populations.

**Table 4:** Sequencing Data

Animal ID	MSH6 Missense	MLH1 Stop codon	MLH1 2 bp deletion
H285	0/1	0/0	0/0
J469	0/1	0/0	0/0
J453	0/1	0/0	0/0
J71	0/1	0/0	0/0
J73	0/1	0/0	0/1
J536	0/0	0/1	0/1
J735	0/0	0/1	0/1
J731	0/0	0/1	0/1
J340	0/0	0/1	0/1
J435	0/0	0/0	0/1
J725	0/0	0/0	0/1
5-101	0/0	0/1	0/1
J737	0/1	0/0	1/1

Genotypes for MMR mutations in CRC macaque cases. 0 = reference allele; 1 = mutation (variant)

To summarize the genetic findings, we identified several different mutations segregating in the KCCMR population that are predicted to negatively affect function of two of the four MMR genes that cause Lynch syndrome in humans, *MLH1* and *MSH6*. The other KCCMR macaques that developed CRC may also carry mutations affecting one or more of the four MMR genes. Such unidentified mutations may fall in enhancers or other regulatory sequences that have not yet been recognized and annotated in the rhesus genome. Any such unrecognized mutations may nevertheless reduce or eliminate expression of functional MMR protein (e.g. promoter or enhancer variants that reduce mRNA expression).

### Pedigree analysis

**Figure 3:**
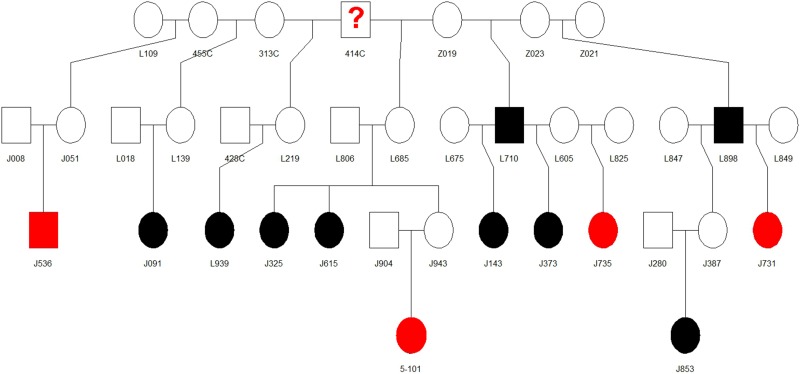
Pedigree for founder 414C taken from the rhesus colony breeding records Shaded animals have definitive diagnosis for CRC. Those shaded black exhibit CRC, but have not been genotyped, while those shaded red have CRC and showed the MLH1 stop codon mutation. Unshaded animals did not show signs of CRC or are still alive in the colony.

Pedigree analysis of the rhesus monkeys with CRC in the KCCMR colony revealed a pattern suggestive of autosomal dominant inheritance with affected animals clustered into certain “family” groups. One such family is seen in Figure 3, four of the animals with stop codon mutations in MLH1 and nine other CRC animals (that have not been genotyped), can be traced back to a common male ancestor, 414C. This common male ancestor for several animals identified with the stop codon mutation in *MLH1* (414C) was considered a “founder male” when the colony was being established in the 1970's. In order to become an SPF colony, the rhesus breeding colony was closed in 1985 to any outside animals. This allowed the original founder males to have a profound influence on colony genetics. Unshaded animals indicate that we do not have definitive evidence of CRC in these animals; however many of these animals were sold or euthanized before reaching geriatric age, and their cause of death or future development of cancer is unknown. For example, the suspected founder male 414C was sold out of the colony and it is unknown whether he eventually developed CRC. Based on the number of descendants with documented mutations, we suspect that he was one of the original carriers of the *MLH1* stop codon mutation.

## DISCUSSION

Rhesus macaques have proven to be a valuable and sometimes essential animal model for various human diseases [19]. Studies of HIV/AIDS, developmental psychobiology, addiction and alcohol abuse, cardiovascular diseases, metabolic diseases and other pathologies have all benefited significantly from research using rhesus macaques as laboratory models. Nonhuman primates do develop spontaneous neoplasias [21-23], but have not been widely used as animal models of cancer. To the best of our knowledge, there are currently no studies employing the rhesus macaque model of CRC to study new therapies for Lynch Syndrome.

The diagnosis of CRC in 60 rhesus macaques over the past 17 years at the KCCMR, with clinicopathologic features that closely mimicked Lynch syndrome in humans, led us to investigate the microsatellite status and MMR protein immunohistochemistry in these 20 animals. There was loss of MLH1 and PMS2 mismatch repair proteins in the majority of tumors and we were also able to demonstrate microsatellite instability in several cases. Pedigree analysis revealed a likely autosomal dominant pattern of inheritance with identification of suspect “founder males” from the creation of the colony in the 1970's. This led us to perform whole genome sequencing on 20 affected animals and compare them to a set of 517 undiagnosed or unaffected rhesus monkeys that we had analyzed for other projects. Several animals within a common lineage were demonstrated to have mutations in *MLH1*. Other affected animals were found to have a missense mutation in *MSH6* that is predicted to be damaging. We did not observe recognizably damaging mutations in *PMS2* in any subject, though this is one of the proteins that showed diminished expression in affected subjects. There may be unrecognized mutations present in DNA sequences that regulate expression of *PMS2*. Alternatively, when the *MLH1* gene is mutated and that protein is reduced or absent, that reduction may lead to instability and loss of its heterodimer partner *PMS2*.

Taken together, these data support the conclusion that CRC in rhesus monkeys at the KCCMR is a novel model of Lynch syndrome. Indeed, one might argue that this is not actually a model of Lynch syndrome but rather is the same disease identified in a different primate species. This rhesus macaque parallel to human Lynch syndrome will be a valuable research resource for a variety of analyses. A spontaneous, naturally occurring primate model of this disorder, caused by mutations in the same MMR genes that lead to Lynch syndrome in humans, will facilitate future studies of pathogenesis, prevention and treatment. One obvious strength of this primate model is that each subject has a fully functional immune system. Although we have not yet examined frameshift mutations and neoantigens in the macaque CRC tumors, we have documented extensive infiltration of lymphocytes into tumors. In addition, access to the macaque reference genome sequence provides information about the similarity of particular protein-coding gene sequences between humans and this nonhuman primate. We found that several genes that display microsatellite mononucleotide runs and frameshift mutations in human MSI positive CRC tumors (e.g. *TGFBR2*, *TAF1B*, *ASTE1* and others) display equivalent mononucleotide runs in the macaques, but not in the orthologous genes in mice. Consequently, the macaques may be susceptible to the equivalent frameshift mutations and formation of neoantigens paralleling those identified in humans.

Tests of novel immunotherapeutic strategies is one obvious application of this macaque model of Lynch Syndrome, as these macaques have intact immune systems that are very similar to human immune systems. Other applications are easily envisioned such as controlled studies designed to investigate the optimal use of aspirin in Lynch syndrome patients. Since mouse models fail to replicate important aspects of human Lynch syndrome [12, 14], we suggest that rhesus macaques with MMR mutations will be significantly more useful in the pursuit of experimental results that translate well to humans.

## MATERIALS AND METHODS

### Pathology

Twenty rhesus macaques that had been diagnosed with a suspected CRC at the MD Anderson Keeling Center for Comparative Medicine and Research over the past 7 years were euthanized due to humane reasons and submitted for necropsy to be evaluated by a board certified veterinary pathologist (BKD, MJM, WBB). The clinical suspicion of CRC was based on a history of weight loss and/or diarrhea, with physical exam often revealing an abdominal mass. A complete necropsy was performed with collection of tissues in 10% neutral buffered formalin. Tissues were processed, embedded, and stained with H&E following standard histopathology protocol. Frozen tissues and blood were collected at the discretion of the pathologist and the clinical veterinarian.

### Immunohistochemistry

Immunohistochemistry (IHC) staining for MLH1, MSH2, MSH6 and PMS2 was performed on formalin fixed paraffin-embedded tumor tissue sections obtained from the 20 affected animals and normal colon from 2 unaffected rhesus monkeys. Tissue sections were cut at 3-4 um and submitted to the Scott and White Medical Center Reference Laboratory in Temple, TX. The following Agilent Dako IHC antibodies were used according to manufacturer's recommendations: MSH6 - IR086 Monoclonal Rabbit Anti-human Muts Protein Homolog 6, clone EP49; MLH1 - IR079, Monoclonal Mouse Anti-human Mutl Protein Homolog 1, clone ES05; PMS2 - IR087, Monoclonal Rabbit Anti-Human Posteiotic Segregation Increase 2, clone EPS1; MSH2 - IR085, Monoclonal Mouse Anti-human Muts Protein Homolog 2, clone FE11.

### Microsatellite instability

Frozen tumor samples and normal tissue (liver, kidney, or normal gastrointestinal tract) from 9 of the rhesus macaques with CRC were available for MSI analysis. We used twelve microsatellite markers, adapted from the National Cancer Institute recommendations [7, 24]. Human intragenic microsatellites BAT-25, BAT-26, and NR-21 were selected as mononucleotide repeat markers to study microsatellite status in tumors from rhesus macaques. Since our previous attempts to amplify rhesus DNA using human dinucleotide markers from the Bethesda panel failed, we selected a set of 9 dinucleotide microsatellites previously described for rhesus macaques [25]: MML3S7, MML3S9, MML4S1, MML11S1, MML14S6, MML16S3, MML17S1, MML17S2, and MML18S3. Markers were PCR amplified using fluorescently-labeled primers (6FAM, VIC, or NED from Applied Biosystems, Foster City, CA) as described elsewhere [26]. The PCR amplifications were performed in an Eppendorf Mastercycler Gradient® instrument (Eppendorf, Mississauga, ON). Samples were loaded in a 96 well plate, denatured at 95°C for 5 min, and analyzed by capillary electrophoresis in an ABI Prism 3130XL instrument (Applied Biosystems) using POP4 polymer (Applied Biosystems). The alleles were visualized and scored with GeneMapper software V. 4.0 (Applied Biosystems). The degree of instability in individual tumor samples was scored according to the number of microsatellite markers showing allele shifts, using a three-part classification: stable (MSI-S, no markers showing instability), low (MSI-L, 1-2 markers showing instability), or high (MSI-H, 3 or more markers showing instability). All samples showing allele shifts were examined three times.

### DNA sequencing

We performed whole genome sequencing using DNA from the 20 macaques found upon necropsy to have CRC, and 7 aged individuals found upon necropsy to be free of CRC. DNA was used to generate standard PCR-free Illumina paired-end sequencing libraries. Libraries were prepared using KAPA Hyper PCR-free library reagents (KK8505, KAPA Biosystems Inc.) in Beckman robotic workstations (Biomek FX and FXp models). We sheared total genomic DNA (500 ng) into fragments of approximately 200-600 bp in a Covaris E220 system (96 well format) followed by purification of the fragmented DNA using AMPure XP beads. A double size selection step was employed, with different ratios of AMPure XP beads, to select a narrow size band of sheared DNA molecules for library preparation. DNA end-repair and 3′-adenylation were then performed in the same reaction followed by ligation of the barcoded adaptors to create PCR-Free libraries, and the library run on the Fragment Analyzer (Advanced Analytical Technologies, Inc, Ames, Iowa) to assess library size and presence of remaining adapter dimers. This was followed by qPCR assay using KAPA Library Quantification Kit using their SYBR® FAST qPCR Master Mix to estimate the size and quantification. These WGS libraries were sequenced on the Illumina HiSeq-X instrument to generate 150 bp paired-end reads. All flow cell data (BCL files) are converted to barcoded FASTQ files, which are then aligned using BWA-mem [27] to the rhesus macaque reference genome (Mmul_8.0.1) to obtain BAM files. Single nucleotide variants (SNVs) and small indels (up to 60bp) were called using GATK software following best practices (https://software.broadinstitute.org/gatk/documentation/). Briefly, the sorted and indexed BAM files are realigned using a known standard indel dataset, base recalibrated, and then joint genotype calling was performed on all samples, resulting in raw genotypes in VCF file format. GATK hard filters (https://software.broadinstitute.org/gatk/documentation/article?id=2806) were applied and variant calls that failed were removed. Using re-sequencing data for the rhesus macaque used to produce the reference assembly (sample ID 17573), any calls in which the reference animal did not have the reference allele were removed.

### Pedigree analysis

The Rhesus Breeding and Research Resource housed at the Michale E. Keeling Center for Comparative Medicine is a closed colony of specific pathogen free (SPF) rhesus. Animals are housed in single-male/multi-female breeding units and pedigrees are kept as part of the animal histories. Complete records of sires and dams were available back to the founding of the colony in the 1970's. These records were examined to determine the relationships between the animals that had been diagnosed with CRC.

## Abbreviations

CRC: Colorectal cancer
MMR: Mismatch repair
TILs: Tumor-infiltrating lymphocytes
HNPCC: Hereditary non-polyposis colorectal cancer
MSI: Microsatellite instability
MNR: Mononucleotide repeats
KCCMR: MD Anderson Cancer Center Keeling Center for Comparative Medicine and Research
